# Anisotropic Failure Strength of Shale with Increasing Confinement: Behaviors, Factors and Mechanism

**DOI:** 10.3390/ma10111310

**Published:** 2017-11-15

**Authors:** Cheng Cheng, Xiao Li, Haitao Qian

**Affiliations:** 1 Key Laboratory of Shale Gas and Geoengineering, Institute of Geology and Geophysics, Chinese Academy of Sciences, Beijing 100029, China; cheng@mail.iggcas.ac.cn; 2 Institutions of Earth Science, Chinese Academy of Sciences, Beijing 100029, China; 3 College of Earth Sciences, University of Chinese Academy of Sciences, Beijing 100049, China; 4 Institute of Crustal Dynamics, China Earthquake Administration, Beijing 100085, China; haitao-qian@163.com

**Keywords:** shale, anisotropic failure, strength anisotropy, confinement, cohesion and friction angle of weak planes

## Abstract

Some studies reported that the anisotropic failure strength of shale will be weakened by increasing confinement. In this paper, it is found that there are various types of anisotropic strength behaviors. Four types of anisotropic strength ratio ($SA_{1}$) behaviors and three types of anisotropic strength difference ($SA_{2}$) behaviors have been classified based on laboratory experiments on nine groups of different shale samples. The cohesion $c_{w}$ and friction angle $\phi_{w}$ of the weak planes are proven to be two dominant factors according to a series of bonded-particle discrete element modelling analyses. It is observed that shale is more prone to a slight increase of $SA_{1}$ and significant increase of $SA_{2}$ with increasing confinement for higher cohesion $c_{w}$ and lower to medium friction angle $\phi_{w}$. This study also investigated the mechanism of the anisotropic strength behaviors with increasing confinement. Owing to different contributions of $c_{w}$ and $\phi_{w}$ under different confinements, different combinations of $c_{w}$ and $\phi_{w}$ may have various types of influences on the minimum failure strength with the increasing confinement; therefore, different types of anisotropic behaviors occur for different shale specimens as the confinement increases. These findings are very important to understand the stability of wellbore and underground tunneling in the shale rock mass, and should be helpful for further studies on hydraulic fracture propagations in the shale reservoir.

## 1. Introduction

It is well known that shale exhibits various degrees of anisotropic failure characteristics and strength values owing to its structures [1,2,3,4]. Anisotropic failure strength, referred to as strength variation with respect to the orientations of principal stresses [4], is of great importance in the stability problems of wellbore in shale gas exploitation and underground tunneling in the shale rock mass [2,5,6]. Extensive studies have been carried out to research the anisotropic strength properties of shale samples from various reservoirs or outcrops [1,2,3,7,8,9,10,11,12,13,14,15]. Several studies also try to build some anisotropic strength criteria which are more reasonable for the shale specimens [2,4,13,16]. In addition, with the rapid development of numerical modelling technology, many different numerical methods have been used to research the anisotropic strength behaviors, such as the finite element method (FEM) [17], discrete element method (DEM) [14,18,19], and the combined finite element method/discrete element method (FEM/DEM) [20], etc. In recent years, the nonlocal lattice particle model has been developed and has proven to be a promising method to analyze anisotropic failure behaviors [21,22].

The shale should be under different stress states at different depths, in different places relative to the underground work, or considering different forms of support after opening. Nonetheless, based on an extensive literature review, no systematic research on the anisotropic strength behaviors of shale with increasing confinement has been found in the previous studies. Some studies reported that the anisotropic failure strength of shale will be weaker with increasing confinement [7,16,23,24]. This empirical understanding is always obtained from the observations on a limited number of test results, based on the anisotropic strength parameters defined as the ratios of strength values at different loading directions [7,11,15,16]. However, is it always correct? It should be noted that the anisotropic strength differences are still quite considerable for the shale samples under higher confinement based on many laboratory experimental results [2,16,23,24,25,26]. It is still necessary to make clear how the anisotropic strength behaviors change for different shale specimens as the confinement increases. Furthermore, what should be the predominant factors, and what is the mechanism for the different anisotropic strength behaviors affected by these factors? These questions should be answered based on a more comprehensive study.

Focusing on the above-mentioned questions, this study tries to give a better understanding of the behaviors, factors and mechanisms of the anisotropic failure strength of shale with increasing confinement. With more detailed analyses on the anisotropic strength parameters to describe the magnitude of strength anisotropy, the classifications of different types of anisotropic strength behaviors are made based on nine groups of laboratory experiments on different shale specimens (Section 2). By bonded-particle discrete element modelling, a series of systematic analyses are conducted to study the influence of the key factors on the anisotropic strength behaviors (Section 3). Based on the well-known Jaeger’s strength criterion, as well as the laboratory and numerical test results, the mechanism of the different anisotropic strength behaviors is discussed in Section 4. This study may help us have a better understanding of the anisotropic strength properties of the shale specimens, especially for wellbore and excavation stability problems, or may even be able to extend to the propagation characteristics of hydraulic fractures in the shale reservoir under different in situ stresses.

## 2. Classifications on Anisotropic Failure Strength Behaviors of Shale by Experimental Results

### 2.1. Degree of Anisotropic Failure Strength

It is of great importance to define suitable parameters to evaluate the degree of anisotropic failure strength. Different parameters have been used in the former studies as listed in Table 1. Although these anisotropic strength parameters have different forms, all of them actually reflect the ratio of strength values at different loading directions. These parameters are dimensionless and have been applied widely for estimating the properties of strength anisotropy.

Here, this method will also be applied in this study and is defined more concisely as

(1) $$SA_{1}=\;\frac{\sigma_{1,\max}}{\sigma_{1,\min}}$$

where $\sigma_{1,\max}$ and $\sigma_{1,\min}$ are the maximum and minimum strengths of shale under the certain confinement, respectively.

Meanwhile, another strength anisotropic parameter $SA_{2}$ is also adopted in this work. It is defined as the difference between the maximum and minimum strength values of shale samples under a certain confinement:

(2) $$SA_{2}=\;\sigma_{1,\max}-\sigma_{1,\min}$$

The physical meanings of the two parameters can be understood based on their definitions. $SA_{1}$ is a dimensionless coefficient from the perspective of strength ratio to evaluate the strength anisotropy at different loading directions. The material can be considered as isotropic for strength if $SA_{1}$ = 1, and a higher value of $SA_{1}$ means a higher degree of anisotropic strength behavior. Nonetheless, $SA_{2}$, with a unit of MPa, shows the specific values of strength differences for the material at different orientations. $SA_{2}$ = 0 MPa refers to the material with isotropic strength, and the increasing value of $SA_{2}$ shows the increasing anisotropic degree of strength. The relation between the two parameters can be described with the following equation:

(3) $$SA_{2}=\;\sigma_{1,\min}\left(SA_{1}-1\right)$$

**Figure 1:**
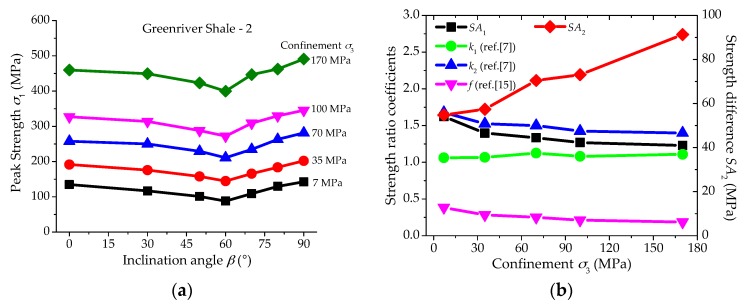
(**a**) Anisotropic strength values of Greenriver Shale-2 samples under various confinements [16], and (**b**) different changing trends of $SA_{1}$, $SA_{2}$ and some other anisotropic parameters with the increasing confinement.

Apparently, with the changing $\sigma_{1,\min}$ under various confinements, $SA_{1}$ and $SA_{2}$ are two independent parameters. The laboratory test results of Greenriver Shale-2 samples [16] are used as an example to show the changing trends of $SA_{1}$ and $SA_{2}$ with the increasing confinements (Figure 1). It should be noted that the inclination angle *β* is defined as the acute angle between the weak planes and the direction of minimum principal stress. The variations of three other anisotropic parameters listed in Table 1 are also plotted in Figure 1b for comparison, while the other two parameters from references [11,27] are not shown here because they only show the anisotropic strength behaviors of the samples under uniaxial compression.

According to Figure 1b, the anisotropic strength ratio $SA_{1}$ decreases with the increasing confinement; however, the anisotropic strength difference $SA_{2}$ shows an increasing trend. Obviously, the reduction of $SA_{1}$ is caused by the increasing $\sigma_{1,\min}$ with the improving confinement. At the meantime, the parameters *k*_2_ and *f* also present a decreasing trend like the parameter $SA_{1}$, because they are all defined from the perspective of strength ratio. The parameter *k*_1_ keeps almost constant because this parameter only considers the strength of the shale samples at the directions parallel and perpendicular to the weak planes, and the anisotropic strength behaviors induced by the structures cannot be reflected completely.

Consequently, $SA_{1}$ and $SA_{2}$ can be used as two typical parameters to demonstrate the degree of anisotropic failure strength from two different perspectives. It is difficult to say which one is better. $SA_{1}$ may be a better parameter to compare the anisotropic strength characteristics of different shale samples because it is a dimensionless coefficient. However, $SA_{2}$ is easier to be applied to estimate the stability of a certain shale based on the strength criterion because it considers the specific values of strength differences. Therefore, both of these two measures should be researched for a systematic and comprehensive understanding on the anisotropic strength behaviors of shale.

### 2.2. Laboratory Experimental Database

A database has been compiled including 251 uniaxial and conventional triaxial compressive tests on shale samples from nine different reservoirs or outcrops. The basic information of the samples and test conditions are presented in Table 2. The first eight groups of test results are collected from the published papers, and more detailed information can be found in the corresponding references if necessary. The last group of experiments are carried out by the authors in this study.

**Figure 2:**
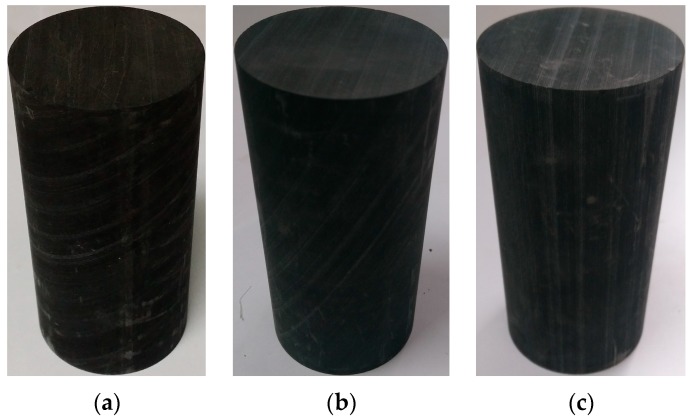
Shale-5 samples with different oriented weak planes. (**a**) *β* = 30°; (**b**) *β* = 60°; and (**c**) *β* = 90°.

The laboratory experiments on Shale-5 specimens are carried out in the MTS815 test machine (Institute of Rock and Soil Mechanics, Chinese Academy of Sciences in Wuhan, China). The intact black shale with a single set of parallel weak planes are well prepared to be cylindrical samples with the size of 50 mm in diameter and 100 mm in height. The samples with various oriented weak planes are shown in Figure 2. The physical and mechanical parameters of the samples are presented in Table 3. It can be observed that Shale-5 samples show various degrees of anisotropic characteristics on P-wave velocity, uniaxial compressive strength, and Young’s modulus.

### 2.3. Different Types of Anisotropic Strength Behaviors Based on SA_1_

**Figure 3:**
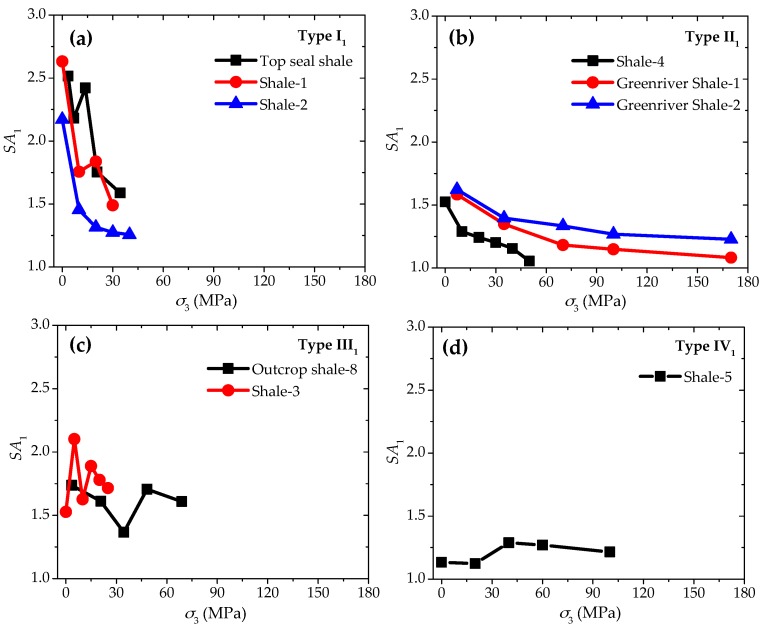
Four types of anisotropic strength behaviors based on *SA*_1_ with increasing confinement. (**a**) Type I_1_; (**b**) Type II_1_; (**c**) Type III_1_; and (**d**) Type IV_1_.

Based on the laboratory test results in the database mentioned above, anisotropic strength ratios $SA_{1}$ are calculated for the different shale samples under different confinements. According to the calculated results exhibited in Figure 3, it is apparent that the anisotropic strength behaviors can be classified into four different types as follows:

(1) Type I_1_: Significant decrease of $SA_{1}$ with increasing confinement.

The anisotropic strength ratio $SA_{1}$ falls significantly as the confinement increases. Taking the Shale-1 samples for example, $SA_{1}$ can be reduced from about 2.63 to 1.49 with *σ*_3_ increasing from 0 to 30 MPa (Figure 3a).

(2) Type II_1_: Slight decrease of *SA*_1_ with increasing confinement.

The anisotropic strength ratio $SA_{1}$ may only be lowered slightly with the confinement going up, for example, $SA_{1}$ of Greenriver Shale-2 decreases from about 1.62 to 1.23 with a high increase of *σ*_3_ from 0 to 170 MPa (Figure 3b).

(3) Type III_1_: Generally constant *SA*_1_ with increasing confinement.

There may be some oscillations of $SA_{1}$ as the confinement increases, but no obvious trend of up or down can be observed. For instance, $SA_{1}$ of Outcrop Shale-8 remains in the range between about 1.37 and 1.74 with some undulations during the rise of *σ*_3_ from 0 to 69 MPa (Figure 3c).

(4) Type IV_1_: Slight increase of *SA*_1_ with increasing confinement.

$SA_{1}$ goes up slightly with the confinement increases. For example, $SA_{1}$ values of Shale-5 are only about 1.12 and 1.13 at the confinement of 0 and 20 MPa, while this ratio increases to 1.29 when *σ*_3_ = 40 MPa. Although there appears a little reduction to 1.27 and 1.21 when *σ*_3_ = 60 and 100 MPa, they are still higher than the cases at *σ*_3_ = 0 and 20 MPa (Figure 3d). 

### 2.4. Different Types of Anisotropic Strength Behaviors Based on SA_2_

**Figure 4:**
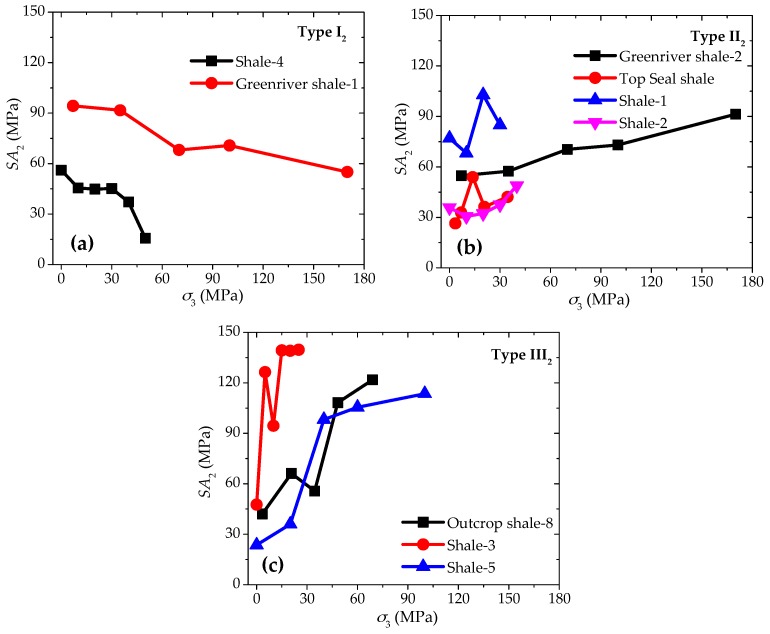
Three types of strength anisotropic behaviors based on *SA*_2_ with increasing confinement. (**a**) Type I_2_; (**b**) Type II_2_; and (**c**) Type III_2_.

The anisotropic strength differences $SA_{2}$ have also been calculated based on the above mentioned nine groups of laboratory experimental results. These $SA_{2}$ values are plotted in Figure 4, and they can be classified into three different types according to their changing trends with increasing confinement:

(1) Type I_2_: Gradual decrease of *SA*_2_ with increasing confinement.

The anisotropic strength difference $SA_{2}$ of this type of shale goes down gradually with the increase of confinement. As an example, $SA_{2}$ of Greenriver Shale-1 goes down gradually from 94.3 to 55.0 MPa with the confinement increases from 0 to 170 MPa (Figure 4a).

(2) Type II_2_: Slight increase of *SA*_2_ with increasing confinement.

For this type of shale samples, $SA_{2}$ value increases much more slightly than that of Type I_2_ with the rise of confinement. Taking Greenriver Shale-2 as an example, $SA_{2}$ goes up gradually from 54.8 to 91.3 MPa with the confinement increases from 0 to 170 MPa (Figure 4b).

(3) Type III_2_: Significant increase of *SA*_2_ with increasing confinement.

With the rise of confinement, there is a significant increase of $SA_{2}$ for this type of shale samples. For example, $SA_{2}$ of Shale-3 increases quickly from 47.6 to 139.7 MPa as the confinement increases from 0 to 30 MPa (Figure 4c).

### 2.5. Discussions

According to the different types of anisotropic strength behaviors of the shale samples based on both of the two parameters $SA_{1}$ and $SA_{2}$, it has been proved that the anisotropic failure strength of shale may have different types of changes with the rise of confinement. With either parameter $SA_{1}$ or $SA_{2}$, there are shale samples with various degrees of increase or decrease as the confinement goes up. It is important to notice these features, and furthermore, it is also important to make clear the dominant factors and how they affect these anisotropic strength behaviors.

Jaeger has proposed a well-known anisotropic strength criterion for the rock containing a set of parallel weak planes [28]:

(4) $$\sigma_{1}=\;\sigma_{3}+\frac{2\left(c_{w}+\sigma_{3}\tan\phi_{w}\right)}{\left(1-\tan\phi_{w}\cot\beta \right)\sin2\beta }$$

where $\sigma_{1}$ and $\sigma_{3}$ are the maximum and minimum principal stresses, β is the acute angel between the weak planes and the direction of minimum principal stress, and $c_{w}$ and $\phi_{w}$ are the cohesion and friction angle of the weak planes, respectively.

According to Jaeger’s strength criterion, it can be seen that $c_{w}$ and $\phi_{w}$ play important roles in the strength of the transversely isotropic rock at different loading directions. For this consideration, they should also have significant influences on the degree of strength anisotropy under different confinements. 

Usually, the shale samples can be considered as intact rock with a set of weak planes. According to the structures of the specimens described in Table 2, it is reasonable and applicable to use Jaeger’s strength criterion to analyze the strength anisotropy of these shale samples. Consequently, $c_{w}$ and $\phi_{w}$ will be considered as two important factors to study their influences on the different anisotropic strength behaviors of shale. For a comprehensive understanding on this problem, numerical modelling will be applied in the next section to make a systematic analysis.

## 3. Anisotropic Strength Behaviors Affected by Cohesion and Friction Angle of Weak Planes

### 3.1. Bonded-Particle Discrete Element Modelling

The bonded-particle discrete element model used in this study is generated by PFC2D (Particle Flow Code in 2 dimensions) developed by Itasca Consulting Group [29]. Parallel bonded particle model is applied for analogue of the rock material, and smooth-joint model is used to simulate the weak planes. 

In the parallel bonded particle model, circular particles are randomly bonded together, and the parallel bonds between the particles have specific strengths and stiffness at the normal and shear directions. The bond will break when each the normal or shear strength is reached, and a crack will be formed at the place of the broken bond. Newton’s second law of motion and a force-displacement law are used to govern the particle movements in each calculation cycle. Thus, a relatively simple set of micro-parameters of the particles and bonds can make the model exhibit emergent macro characteristics including fracture propagation, strength behaviors, dilation, strain hardening and softening, etc. [14,29,30,31,32,33].

With the introduction of the smooth-joint model, the simulation of structural planes can avoid the inherent roughness arising from the circular shape of the particles, because the particles can move along the direction of the structural planes, instead of having to rotate the other balls. In addition, in the smooth-joint model, the properties such as cohesion, friction angle, dilation angle etc. can be set directly to the weak planes, and it is very convenient to study the effects of these parameters on the macro behaviors of the rock [34,35,36,37,38].

A more detailed theory and algorithm can be found in the references mentioned above. By using the parallel bonded particle model incorporated with the smooth-joint model, some research has successfully simulated the strength and deformation behaviors of shale samples [14], as well as the hydraulic fracturing process in the reservoir [39].

**Figure 5:**
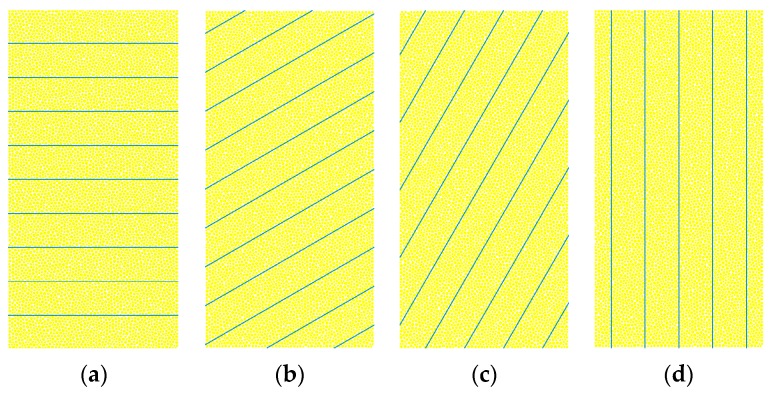
Numerical models for the shale samples with different oriented weak planes. (**a**) *β* = 0°; (**b**) *β* = 30°; (**c**) *β* = 60°; and (**d**) *β* = 90°.

**Figure 6:**
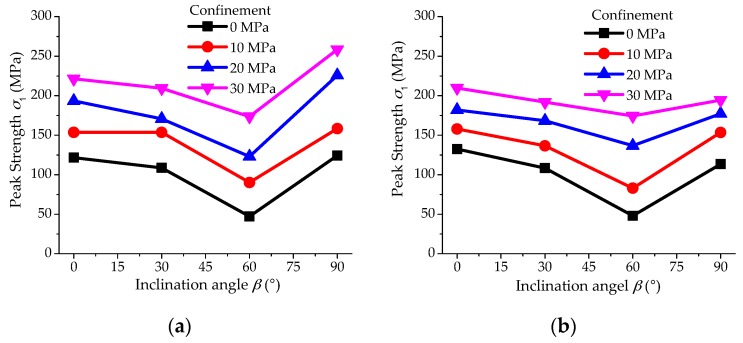
(**a**) The peak strength values of Shale-1 samples at different loading directions under various confinements [25]; and (**b**) the validated strength values of the numerical model.

Consequently, it is suitable to select this modelling method for the analysis of anisotropic failure strength here. In this study, there is only a single set of weak planes in the samples, and a 2D model can give reasonable analogue of the strength behaviors of the shale specimens; besides, it can save much more computing time than the 3D model. Consequently, a 2D model is applied in this study. The validation of the numerical model is based on the test results of Shale-1 samples [25]. The model with 6250 particles has a size of 50 mm in diameter and 100 mm in height (Figure 5). The validated micro-parameters for the bonded particle model and smooth-joint model are presented in Table 4 and Table 5, respectively. The tested and modelled anisotropic strength values are presented in Figure 6a,b, respectively. It can be found that this model can generally simulate the various strength values of the Shale-1 samples with different oriented weak planes under different confinements. Although the modelled strength values are a little lower at *β* = 90° under the confinement $\sigma_{3}$ = 20 and 30 MPa, the model is reasonable enough to study the trends of anisotropic strength behaviors and mechanism of shale in this work.

### 3.2. Modelling Analyses

In this study, the modelling shale samples containing weak planes with four different inclination angles *β* = 0°, 30°, 60°, and 90° will be investigated under four different confinements $\sigma_{3}$ = 0, 10, 20 and 30 MPa. Different combinations of cohesion $c_{w}$ = 10, 20 and 40 MPa as well as friction angle $\phi_{w}$ = 10°, 30°, and 50° will be considered for the weak planes. Influence of cohesion and friction angle of weak planes will both be studied in the following analyses. 

#### 3.2.1. Influence of Cohesion of Weak Planes

**Figure 7:**
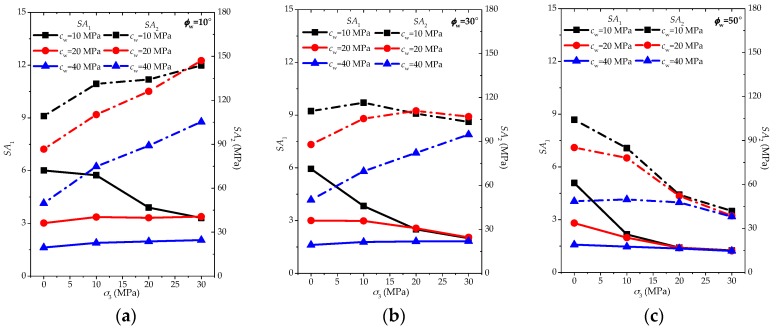
Influence of cohesion of weak planes with the certain friction angle (**a**) *φ*_w_ = 10°; (**b**) *φ*_w_ = 30°; and (**c**) *φ*_w_ = 50° based on *SA*_1_ (solid line) and *SA*_2_ (dashed line).

Based on the modelling results, the anisotropic strength parameters $SA_{1}$ and $SA_{2}$ are plotted in Figure 7 to obtain an understanding on the influence of weak plane cohesion $c_{w}$ on the anisotropic behaviors.

According to Figure 7, several observations on the variations of $SA_{1}$ with increasing confinement can be obtained as follows:

(1) In the case of a low to medium friction angle ($\phi_{w}$ = 10° and 30°), the increase of cohesion $c_{w}$ may transfer the $SA_{1}$ behaviors from significant decrease to slight decrease or even slight increase with the confinement going up;
(2) For a high friction angle ($\phi_{w}$ = 50°), increasing cohesion $c_{w}$ can also change the $SA_{1}$ features from significant decrease to slight decrease, however, it is difficult to obtain the increasing trend of $SA_{1}$ with the rise of confinement;
(3) Generally speaking, the lower cohesion $c_{w}$ may be prone to lead to the significant decrease of $SA_{1}$ with the increasing confinement, while the higher cohesion $c_{w}$ will weaken this trend, but whether it will be slight decrease or increase is dependent on the friction angle $\phi_{w}$ of the weak planes.

Meanwhile, it is not difficult to find some features of $SA_{2}$ with increasing confinement:

(1) When friction angle $\phi_{w}$ = 10°, the increase of cohesion $c_{w}$ may lower all $SA_{2}$ values under various confinements, and the increasing trend of $SA_{2}$ will be more significant with the increasing confinement;
(2) When friction angle $\phi_{w}$ = 30°, the increase of cohesion $c_{w}$ makes the slight decreasing trend of $SA_{2}$ transfer to a slight or significant increase with the increasing confinement;
(3) When friction angle $\phi_{w}$ = 50°, the increase of cohesion $c_{w}$ makes the significant decreasing trend of $SA_{2}$ transfer to a slight decrease as the confinement increases.

Comparing with the features of $SA_{1}$, there are more cases of increasing trend for $SA_{2}$. Nonetheless, for both parameters $SA_{1}$ and $SA_{2}$, it is similar that the increase of cohesion $c_{w}$ may be prone to weaken the degree of the decreasing trend or transfer it to slight increasing behaviors.

#### 3.2.2. Influence of Friction Angle of Weak Planes

**Figure 8:**
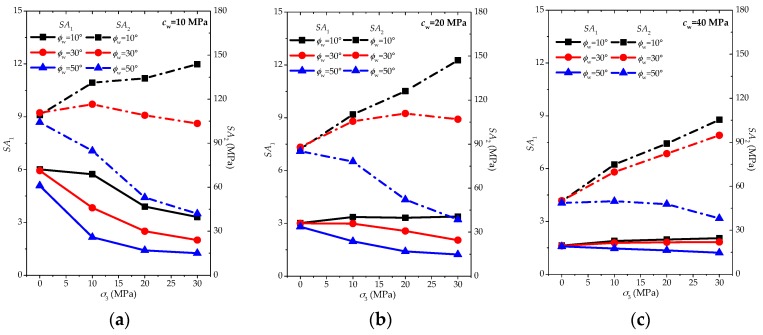
Influence of friction angle of weak planes with the certain cohesion (**a**) *c*_w_ = 10 MPa; (**b**) *c*_w_ = 20 MPa; and (**c**) *c*_w_ = 40 MPa based on *SA*_1_ (solid line) and *SA*_2_ (dashed line).

In order to obtain an understanding of the influence of friction angle $\phi_{w}$ on the anisotropic strength behaviors, the parameters $SA_{1}$ and $SA_{2}$ are again plotted in Figure 8 for another series of comparative studies.

According to Figure 8, the changing trend of $SA_{1}$ with the increasing confinement can be easily observed as follows:

(1) For lower cohesion ($c_{w}$ = 10 MPa), the increasing friction angle $\phi_{w}$ can make the decreasing trend of $SA_{1}$ more and more significant;
(2) For medium to higher cohesion ($c_{w}$ = 20 and 40 MPa), the increasing friction angle $\phi_{w}$ may transfer the slight increasing or almost constant trend of $SA_{1}$ to slight increasing behaviors;
(3) As the cohesion $c_{w}$ increases, the influence of friction angle $\phi_{w}$ on the degree of $SA_{1}$ changing behaviors is more and more limited.

Generally speaking, lower friction angle $\phi_{w}$ is prone to result in the slight decrease or even slight increase of $SA_{1}$, while higher friction angle $\phi_{w}$ may easily induce the slight or even significant decrease of $SA_{1}$ with the confinement going up.

We can observe the features of $SA_{2}$ with the increasing confinement as follows:

(1) For all cases of cohesion ($c_{w}$ = 10, 20 and 40 MPa), the increasing friction angle $\phi_{w}$ can induce the transferring of the $SA_{2}$ trend from going up to going down with the rise of confinement;
(2) As the cohesion $c_{w}$ increases, the influence of friction angle $\phi_{w}$ on the degree of $SA_{2}$ changing behaviors is more and more limited.

Generally speaking, for both parameters $SA_{1}$ and $SA_{2}$, lower friction angle $\phi_{w}$ is prone to result in the slight decrease or even slight increase of anisotropic strength behaviors, while higher friction angle $\phi_{w}$ may easily induce the slight or even significant decrease of anisotropic strength behaviors with the rise of confinement.

#### 3.2.3. Conjoint Analysis on Both Factors $c_{w}$ and $\phi_{w}$

According to the numerical modelling analyses considering various combinations of $c_{w}$ and $\phi_{w}$ mentioned above, the anisotropic strength features covers all the four types of $SA_{1}$ behaviors and three types of $SA_{2}$ behaviors presented in Section 2 based on laboratory experimental results. Here, the types of all numerical cases are plotted in Table 6 to have a better understanding on the influences of $c_{w}$ and $\phi_{w}$ on the anisotropic strength behaviors with increasing confinement.

Table 6 presents various types of $SA_{1}$ behaviors for all combinations of $c_{w}$ and $\phi_{w}$. It is more apparent to find that the increase of cohesion $c_{w}$ will weaken the decreasing trend of $SA_{1}$ from Type I_1_ (significant decrease) to II_1_ (slight decrease), III_1_ (generally constant), or even IV_1_ (slight increase). Meanwhile, the cases with lower friction angle $\phi_{w}$ are more prone to have weaker decreasing trend of $SA_{1}$ or even increase of $SA_{1}$. The phenomenon of increasing $SA_{1}$ with increasing confinements occurs for the cases with lower to medium friction angle ($\phi_{w}$ = 10° and 30°) and higher cohesion ($c_{w}$ = 40 MPa).

Three types of $SA_{2}$ behaviors for all combinations of $c_{w}$ and $\phi_{w}$ are plotted in Table 6. It is also found that it is more prone to have significant increase of $SA_{2}$ for the cases with lower friction angle. With increasing $\phi_{w}$, $SA_{2}$ changes from Type III_2_ (significant increase) to II_2_ (slight decrease), or I_2_ (significant decrease). What is more, the medium to higher cohesion $c_{w}$ is more probable to induce significant increase of $SA_{2}$ with the increasing confinement.

**Figure 9:**
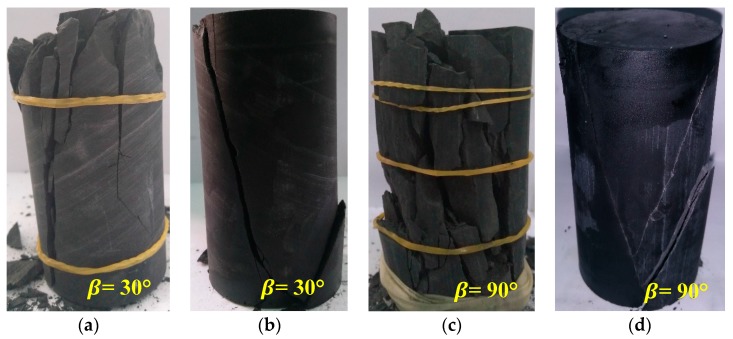
Different failure patterns of Shale-5 samples with inclination angle β = 30° under the confinements of (**a**) *σ*_3_ = 0 MPa; (**b**) *σ*_3_ = 60 MPa and β = 90° under the confinements of (**c**) *σ*_3_ = 0 MPa; (**d**) *σ*_3_ = 60 MPa.

The tests on Shale-5 samples can be applied here as examples to examine the above-mentioned analyses. As shown in Figure 3d and Figure 4c, Shale-5 samples have Type IV_1_ (slight increase) for $SA_{1}$ behavior and Type III_2_ (significant increase) for $SA_{2}$ behavior. The fracturing patterns are found to be closely related to the strength characteristics of the samples and the properties of the weak planes [40,41]. Figure 9 presents the typical failure patterns of Shale-5 samples with different inclination angles (β = 30° and 90°) under different confinements (*σ*_3_ = 0 and 60 MPa). For the case of β = 30° (Figure 9a,b), the specimen mainly fails by vertical extension fractures in the shale matrix under uniaxial compression (*σ*_3_ = 0 MPa), while shear failure planes can be observed crossing the weak planes under the confinement of *σ*_3_ = 60 MPa. No obvious sliding can be observed along the weak planes, and the failure is mainly controlled by the strength of the shale matrix. For the case of β = 90° (Figure 9c,d), the failure takes place by vertical extension along the weak planes under uniaxial compression (*σ*_3_ = 0 MPa), and by shear fractures in the shale matrix under the confinement of *σ*_3_ = 60 MPa. For both of these two cases, the strength of Shale-5 samples are not significantly weakened by the weak planes.

**Figure 10:**
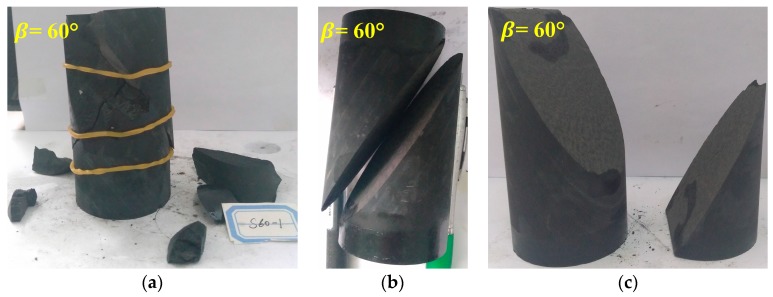
Different failure patterns of Shale-5 samples with inclination angle β = 60° under the confinements of (**a**) *σ*_3_ = 0 MPa; (**b**) *σ*_3_ = 60 MPa and (**c**) *σ*_3_ = 100 MPa.

**Figure 11:**
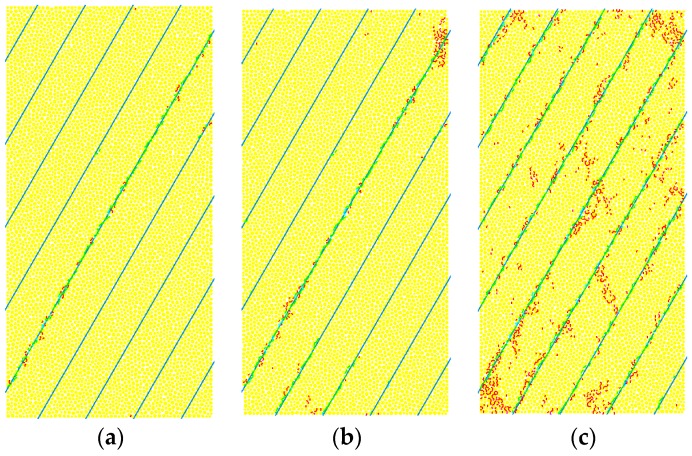
Different fracture characteristics of the samples with inclination angle β = 60° under confinement of 30 MPa by numerical simulations. (**a**) *c*_w_ = 40 MPa, *φ*_w_ = 10°; (**b**) *c*_w_ = 40 MPa, *φ*_w_ = 30°; and (**c**) *c*_w_ = 40 MPa, *φ*_w_ = 50°. Blue color shows the position of the weak planes; Red and magenta colors show the tensile and shear micro-cracks in the matrix; Cyan and green colors show the tensile and shear micro-cracks in the weak planes.

However, the weak planes have different degrees of influences on the strength of Shale-5 samples with inclination angle β = 60°. The fracturing patterns of the specimens are exhibited in Figure 10. It is observed that there are both failures along the weak planes and fractures in the rock material under the confinement *σ*_3_ = 0 MPa; however, the failure is totally along the weak planes, and the fracture surface is very flat and quite smooth under higher confinement *σ*_3_ = 20, 40, 60 and 100 MPa. These fracturing characteristics show that the weak planes of Shale-5 samples have high cohesion $c_{w}$ but relatively low friction angle $\phi_{w}$. This estimation can be supported by the numerical results shown in Figure 11. For the numerical samples with β = 60° and $c_{w}$ = 40 MPa, different values of $\phi_{w}$ result in different failure characteristics under the confinement *σ*_3_ = 30 MPa. For lower $\phi_{w}$ = 10°, the failure mainly slips along the weak planes. As $\phi_{w}$ increases to 30°, a few cracks can be observed in the shale matrix. When $\phi_{w}$ is as high as 50°, there are lots of fractures shown in the shale matrix. Although this numerical model is not exactly the same with the conditions of Shale-5 samples, it can demonstrate that lower $\phi_{w}$ may result in slip along the weak planes but higher $\phi_{w}$ may induce the fractures in the shale matrix for the samples with β = 60° under high confinements. 

In fact, as the fracture is along the weak planes under higher confinement, we can obtain the normal and shear stresses (*σ*_n_ and *τ*) on the weak planes by the stress transformation equations:

(5) $$\sigma_{n}=\;\frac{1}{2}(\sigma_{1}+\sigma_{3})+\frac{1}{2}(\sigma_{1}-\sigma_{3})\cos2\beta$$

(6) $$\tau =\;\frac{1}{2}(\sigma_{1}-\sigma_{3})\sin2\beta$$

**Figure 12:**
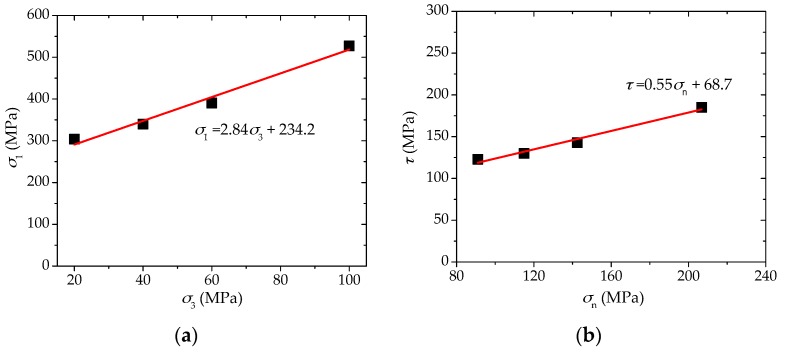
(**a**) Peak strengths of Shale-5 sample (β = 60°) under various confinements (*σ*_3_ = 20, 40, 60, and 100 MPa); and (**b**) normal and shear stresses on the weak planes based on the data in (**a**) and the linearly fitted equation.

Based on the peak strength values of Shale-5 sample (β = 60°) under various confinements (*σ*_3_ = 20, 40, 60 and 100 MPa) presented in Figure 12a, the normal and shear stresses on the weak planes can be calculated according to Equations (5) and (6), and they are plotted in Figure 12b. According to Coulomb’s criterion for structural planes:

(7) $$\tau =\;\sigma_{n}\tan\phi_{w}+c_{w}$$

The cohesion $c_{w}$ and friction angle $\phi_{w}$ of Shale-5 samples can be obtained from the linearly fitted equation in Figure 12b as $c_{w}$ = 68.7 MPa and $\phi_{w}$ = 28.8°. This means that Shale-5 samples have a high cohesion $c_{w}$ and medium to lower friction angle $\phi_{w}$ of the weak planes. Considering that Shale-5 samples have Type IV_1_ (slight increase) for $SA_{1}$ behavior and Type III_2_ (significant increase) for $SA_{2}$ behavior, it is consistent with the analyses by the numerical results that it is more prone to have slight increase of $SA_{1}$ and significant increase of $SA_{2}$ with increasing confinement for medium to higher cohesion $c_{w}$ and lower to medium friction angle $\phi_{w}$.

## 4. Discussions

Based on a series of laboratory experimental results presented in Section 2, it is found that different shale samples may show different anisotropic strength behaviors with increasing confinement, and they can be classified into different types with two anisotropic strength parameters $SA_{1}$ and $SA_{2}$. According to the numerical analyses in Section 3, it has been proved that the cohesion and friction angle of the weak planes indeed have predominant influences on the variation of strength anisotropy of the shale samples. However, it is still necessary to make clear the mechanism of these influences.

According to Jaeger’s strength criterion in Equation (3), the maximum strength $\sigma_{1,\max}$ reaches at β ≤ $\phi_{w}$ or β = 90°, and the value is almost equal to the strength of rock material. The minimum strength $\sigma_{1,\min}$ occurs at $\beta =\frac{\pi }{4}+\frac{\phi_{w}}{2}$, and can be deduced as [42]:

(8) $$\sigma_{1,\min}=\;\sigma_{3}+2\left(c_{w}+\mu_{w}\sigma_{3}\right)\left[{\left(1+\mu_{w}^{2}\right)}^{\frac{1}{2}}+\mu_{w}\right]$$

where, $\mu_{w}=\tan\phi_{w}$.

Consequently, the degree of strength anisotropy is mainly related to the values of minimum strength $\sigma_{1,\min}$. According to Equation (8), cohesion $c_{w}$ plays a role independent of confinement, while the effect of friction angle $\phi_{w}$ is closely related to the confinement *σ*_3_. Under lower confinement, friction angle $\phi_{w}$ has very limited influences on the strength, so cohesion $c_{w}$ becomes more important here. As the confinement goes up, the role of friction angle $\phi_{w}$ with different values may have different degrees of enhancing, while the effect of cohesion $c_{w}$ may not be improved significantly. Consequently, different combinations of $c_{w}$ and $\phi_{w}$ may have various types of influences on the minimum strength $\sigma_{1,\min}$ with the increasing confinement *σ*_3_. Thereafter, different types of anisotropic strength behaviors can be shown for different shale samples with increasing confinement.

**Figure 13:**
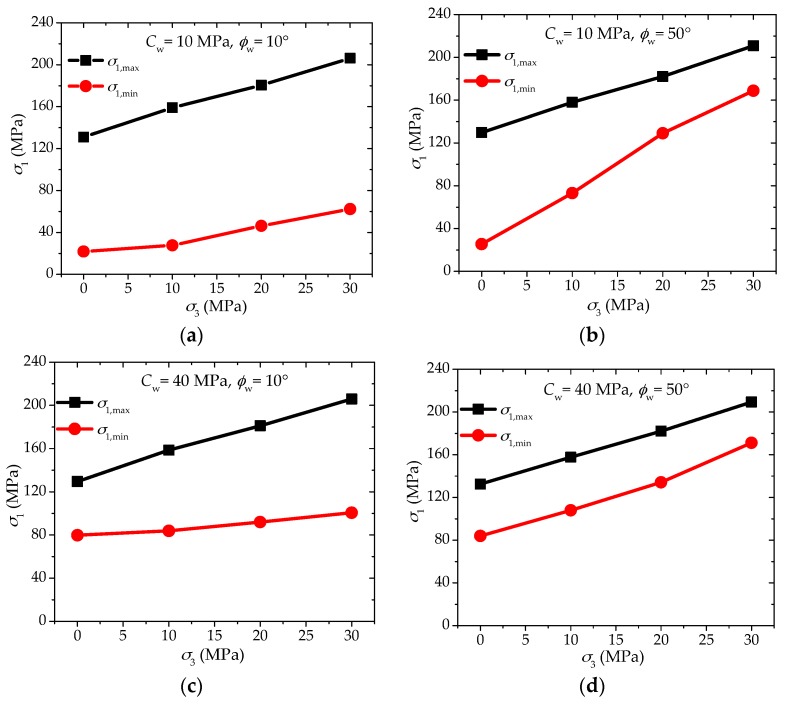
Four typical behaviors of maximum and minimum strengths with increasing confinement dominated by different combinations of cohesion $c_{w}$ and friction angle $\phi_{w}$ of the weak planes based on numerical analyses. (**a**) *c*_w_ = 10 MPa, *φ*_w_ = 10°; (**b**) *c*_w_ = 10 MPa, *φ*_w_ = 50°; (**c**) *c*_w_ = 40 MPa, *φ*_w_ = 10°; and (**d**) *c*_w_ = 40 MPa, *φ*_w_ = 50°.

In order to give a clearer explanation, four combinations of $c_{w}$ (10 and 40 MPa) and $\phi_{w}$ (10° and 50°) are selected from the PFC2D modelling results, and four typical features of the maximum and minimum strength of the shale samples can be observed as follows (Figure 13):

(1) Case I: for lower cohesion ($c_{w}$ = 10 MPa) and lower friction angle ($\phi_{w}$ = 10°), there is quite a large difference between $\sigma_{1,\max}$ and $\sigma_{1,\min}$ under lower confinement mainly resulted from the low value of $c_{w}$, and the strength difference is also very considerable under higher confinement because the low value of $\phi_{w}$ cannot increase $\sigma_{1,\min}$ effectively with the increasing *σ*_3_. In this case, the anisotropic strength ratio $SA_{1}$ may be lowered with increasing confinement, while the anisotropic strength difference $SA_{2}$ may not increase or decrease significantly.
(2) Case II: for lower cohesion ($c_{w}$ = 10 MPa) and higher friction angle ($\phi_{w}$ = 50°), the difference between $\sigma_{1,\max}$ and $\sigma_{1,\min}$ is again very large under lower confinement owing to the low $c_{w}$, however, as the high value of $\phi_{w}$ can enhance $\sigma_{1,\min}$ significantly under higher confinement, the strength difference turns much smaller. In this case, both anisotropic strength ratio $SA_{1}$ and anisotropic strength difference $SA_{2}$ will decrease obviously with the increase of confinement.
(3) Case III: for higher cohesion ($c_{w}$ = 40 MPa) and lower friction angle ($\phi_{w}$ = 10°), the difference between $\sigma_{1,\max}$ and $\sigma_{1,\min}$ is much smaller than the first two cases as the cohesion $c_{w}$ has quite a high value, while the strength difference becomes larger with the increasing confinement because the low value of $\phi_{w}$ leads to quite a low $\sigma_{1,\min}$. In this case, the anisotropic strength ratio $SA_{1}$ may remain almost constant or ever increase slightly with the increasing confinement, while the anisotropic strength difference $SA_{2}$ will increase significantly.
(4) Case IV: for higher cohesion ($c_{w}$ = 40 MPa) and higher friction angle ($\phi_{w}$ = 50°), there is quite a small difference between $\sigma_{1,\max}$ and $\sigma_{1,\min}$ under lower confinement attributed to the high value of $c_{w}$, and the strength difference is also very limited under higher confinement because the high value of $\phi_{w}$ can increase $\sigma_{1,\min}$ effectively with the increasing *σ*_3_. Similar to the first case, the anisotropic strength ratio $SA_{1}$ may be lowered with increasing confinement, while the anisotropic strength difference $SA_{2}$ may not change significantly.

**Figure 14:**
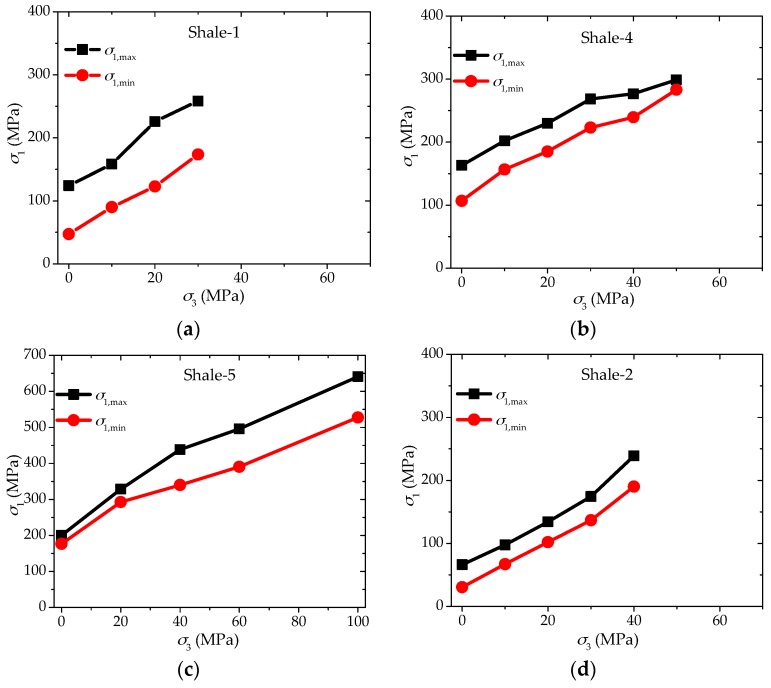
Four typical behaviors of maximum and minimum strengths with increasing confinement based on laboratory experimental results of (**a**) Shale-1; (**b**) Shale-4; (**c**) Shale-5; and (**d**) Shale-2.

It is not difficult to find examples from the laboratory experimental results corresponding with the four typical cases mentioned above. Four such examples are presented in Figure 14. This proves that the mechanism on the anisotropic failure strength behaviors of shale with increasing confinement in this study is reasonable.

It is very important to understand this mechanism when dealing with problems such as the wellbore stability in the shale reservoir. Under different in situ stresses, the shale reservoir with different combinations of $c_{w}$ and $\phi_{w}$ may show different types of anisotropic failure strength behaviors, which is related to the failure patterns of the wellbore. $c_{w}$ and $\phi_{w}$ of the shale may be related to the mineral contents, alignment of the minerals, the geometrical and mechanical properties of the natural fractures, etc., which requires further studies in future work.

## 5. Conclusions

According to a series of systematic analyses on the laboratory test results of nine groups of different shale samples, this work studied the various types of anisotropic failure strength behaviors of shale with increasing confinement, using two different anisotropic strength parameters. In addition, the dominant factors and the mechanism have also been studied combining the test results with numerical analyses. There are several main findings as follows:

(1) Two anisotropic strength parameters, $SA_{1}$ from the perspective of strength ratio and $SA_{2}$ from the perspective of strength difference, should both be researched for a comprehensive understanding of the anisotropic strength behaviors of shale under different confinements. $SA_{1}$ is better for comparing the anisotropic strength characteristics of different shale samples as a dimensionless coefficient, while $SA_{2}$ is easier to be applied to estimate the stability of a certain shale based on the strength criterion because it considers the specific values of strength differences;
(2) Based on the laboratory experimental results of nine groups of different shale samples, it is found that there are four types of $SA_{1}$ behaviors (significant decrease, slight decrease, generally constant, and slight increase) and three types of $SA_{2}$ behaviors (gradual decrease, slight increase, and significant increase) with increasing confinement;
(3) With the parallel bonded particle model simulating the rock material and smooth-joint model simulating the weak planes, the different types of anisotropic strength behaviors are well reproduced in the numerical models. By a series of systematic analyses, it is observed that cohesion $c_{w}$ and friction angle $\phi_{w}$ of the weak planes are two dominant factors for the anisotropic strength behaviors;
(4) The increase of cohesion $c_{w}$ will change the $SA_{1}$ behaviors from significant decrease to slight decrease with increasing confinement, or even slight increase if the friction angle $\phi_{w}$ is medium to low. Meanwhile, the decrease of friction angle $\phi_{w}$ are more prone to transfer $SA_{2}$ behaviors from gradual decrease to slight increase with increasing confinement, or even significant increase if the cohesion $c_{w}$ is medium to high;
(5) The mechanism of the anisotropic strength behaviors have been analyzed based on the well-known Jaeger’s strength criterion, as well as the laboratory and numerical test results. Under lower confinement, cohesion $c_{w}$ has more important roles as the friction angle $\phi_{w}$ has very limited influences on the strength. As the confinement goes up, the friction angle $\phi_{w}$ with different values may take different degrees of roles, while the effect of cohesion $c_{w}$ is not easy to be improved significantly. Consequently, different combinations of $c_{w}$ and $\phi_{w}$ may have various types of influences on the minimum failure strength with the increasing confinement, therefore different shale samples show different types of anisotropic behaviors with the increasing confinement.

It should be noted that these findings are based on the two proposed anisotropic parameters $SA_{1}$ and $SA_{2}$, while there are also some other measures used in other studies. This study has analyzed the relation among these different measures, and it is shown that $SA_{1}$ and $SA_{2}$ are two typical parameters from two different perspectives. Consequently, these findings are reasonable and important in order to have a comprehensive understanding of the behaviors, factors, and mechanism of anisotropic strength of shale under different confining pressures. This understanding should be helpful in guiding the design and construction of the wellbore drilling and underground opening in the rock mass of shale. As an extension, this work should also be useful for understanding the propagations of hydraulic fractures in shale reservoirs under different stress states. This should be studied further based on the mechanism proposed in this work.

## Figures and Tables

**Table 1:** Different parameters to describe the degree of anisotropic failure strength.

Parameters	Descriptions	References
$k_{1}=\;\frac{{\left(\sigma_{1}-\sigma_{3}\right)}_{∥}}{{\left(\sigma_{1}-\sigma_{3}\right)}_{⊥}}$	Ratio between the failure stresses in the two principal directions parallel and perpendicular to the bedding planes, respectively	[7]
$k_{2}=\;\frac{{\left(\sigma_{1}-\sigma_{3}\right)}_{\max}}{{\left(\sigma_{1}-\sigma_{3}\right)}_{\min}}$	Ratio of the maximum to minimum failure strengths	[7]
$\sigma_{c}\left(\max\right)/\sigma_{c}\left(\min\right)$	Ratio of the maximum to minimum uniaxial compressive strength (UCS)	[11]
$f=\;\frac{\sigma_{1,\max}-\sigma_{1,\min}}{\sigma_{1,\max}}$	Ratio of the strength difference to the maximum strength	[15]
$R_{c}=\sigma_{ci\left(90\right)}/\sigma_{ci\left(\min\right)}$	Ratio between the UCS perpendicular to the beddings and the minimum UCS	[27]

**Table 2:** Basic information of the samples and test conditions.

Samples	Description	Inclination Angle *β* (°)	Confinement (MPa)	Ref.
Greenriver shale-1	Light brown to light gray; highly laminated, composed of fine grained calcite and dolomite particles inter-bedded with kerogen	0, 15, 20, 30, 45, 60, 75, 90	7, 35, 70, 100, 170	[16]
Greenriver shale-2	Much darker, with more oil; highly laminated, composed of fine grained calcite and dolomite particles inter-bedded with kerogen	0, 10, 20, 30, 40, 60, 90	7, 35, 70, 100, 170	[16]
Outcrop shale-#8	Gray to dark, with obvious plane of anisotropy shown in the photographs	0, 15, 30, 45, 60, 75, 90	3, 21, 35, 48, 69	[2]
Top seal shale	-	0, 15, 30, 45, 60, 75, 90	3, 7, 14, 21, 35	[2]
Shale-1	Black shale from outcrop of Longmaxi Formation in China, with laminated structures from the SEM images	0, 30, 60, 90	0, 10, 20, 30	[25]
Shale-2	Cored black shale (3502.61~3508.63 m deep) of Longmaxi Formation in Sichuan, China, with planes of anisotropy	0, 15, 30, 45, 60, 75, 90	0, 10, 20, 30, 40	[24]
Shale-3	Black shale at the lower part of Longmaxi Formation in Guizhou, China, with laminated structures and micro-fissures from the SEM images	0, 45, 90	0, 5, 10, 15, 20, 25	[26]
Shale-4	Black shale of Niutitang Formation in China, showing obvious sedimentary rock feature from micrometer scale, with lamellar minerals	0, 30, 45, 60, 90	0, 10, 20, 30, 40, 50	[23]
Shale-5	Black shale from outcrop of Longmaxi Formation in Chongqing, China, with visible planes of anisotropy	30, 60, 90	0, 20, 40, 60, 100	This study

**Table 3:** Basic physical and mechanical properties of Shale-5 samples.

Inclination Angle *β* (°)	*V*p (m/s)	UCS (MPa)	*E* (GPa)
30	4370	191.3	29.8
60	4706	176.6	32.7
90	4964	200.2	34.5

**Table 4:** Validated micro-parameters of the parallel bonded particle model.

Grain (Particles)		Cement (Parallel Bonds)	
Ball density (kg/m^3^)	2700	Bond modulus ${\bar{E}}_{c}$ (GPa)	21
Minimum ball radius (mm)	0.36	Normal bond strength (MPa)	90
Ball radius ratio *R*_max_/*R*_min_	1.66	S.D. ^1^ normal bond strength (MPa)	15
Contact modulus *E*_c_ (GPa)	21	Shearing bond strength (MPa)	90
Coefficient of friction	1.0	S.D. ^1^ shearing bond strength (MPa)	15
Normal to shearing stiffness ratio *k*_n_/*k*_s_	2.5	Normal to shearing bond stiffness ratio ${\bar{k}}_{n}$/${\bar{k}}_{s}$	2.5

^1^ S.D.: standard deviation.

**Table 5:** Validated parameters of the smooth-joint model.

Parameters	Values
Cohesion *C*_sj_ (MPa)	20
Friction angle *φ*_j_ (°)	50
Dilation angle *ψ*_j_ (°)	0
Normal stiffness *k*_n,sj_ (GPa/m)	1500
Shear stiffness *k*_s,sj_ (GPa/m)	2500
Tensile strength *σ*_n,sj_ (MPa)	5

**Table 6:** Influence of cohesion and friction angle of weak planes on *SA*_1_, and *SA*_2_ behaviors.

SA_1_				SA_2_			
	$\phi_{w}$ = 10°	$\phi_{w}$ = 30°	$\phi_{w}$ = 50°		$\phi_{w}$ = 10°	$\phi_{w}$ = 30°	$\phi_{w}$ = 50°
$c_{w}$ = 10 MPa	I_1_	I_1_	I_1_	$c_{w}$ = 10 MPa	II_2_	I_2_	I_2_
$c_{w}$ = 20 MPa	III_1_	II_1_	II_1_	$c_{w}$ = 20 MPa	III_2_	II_2_	I_2_
$c_{w}$ = 40 MPa	IV_1_	IV_1_	II_1_	$c_{w}$ = 40 MPa	III_2_	III_2_	I_2_
